# Beyond antibiotics: the expanding horizon of microbial natural products

**DOI:** 10.3389/frabi.2026.1768331

**Published:** 2026-02-26

**Authors:** Suchitra Ku Panigrahy, Amrita kumari Panda, Aseem Kerketa, Rojita Mishra

**Affiliations:** 1 Department of Biotechnology, Faculty of Science, Kalinga University, Raipur, CG, India; 2 Department of Biotechnology, Sant Sant Gahira Guru Vishwavidyalaya, Ambikapur, CG, India; 3 Department of Botany, Polasara Science College, Polsara,, Odisha, India

**Keywords:** antibiotics, antibiotics resistance, antimicrobial peptides, bacteriocins, microbial modulation

## Abstract

The continuous use of antibiotics has led to the development of antibiotic resistance among bacterial pathogens, posing a significant threat to both human and animal health. This necessitates exploring alternative solutions to combat this growing resistance. Natural products offer a viable alternative for microbial modulation, exhibiting diverse antibacterial processes and the capacity to modify microbial communities and biofilms. These compounds show potential as supplementary agents against resistant infections. Natural products derived from microbes are utilized as biofertilizers and biopesticides, enhancing crop yield and controlling plant pathogens, thereby offering an eco-friendly alternative to chemical fertilizers. Antimicrobial peptides (AMPs) are crucial for combating fish-associated pathogens, reducing mortality rates in the aquaculture industry. Various bacteriocins, are used as food preservatives to inhibit spoilage and pathogenic microorganisms proving their potential in the food industry. In this review, the potential role of natural products from microbes in the food, agriculture, and aquaculture industry sectors has been elucidated. The challenges and prospects were also discussed to provide a foundation for identifying new research opportunities.

## 1 Introduction

The extensive use of antibiotics for decades has led to a significant issue of antibiotic resistance among bacterial pathogens, posing a serious threat to both human and animal health (Seal et al., 2018). This necessitates the exploration of alternative solutions to combat this growing resistance (Seal et al., 2018). Natural products offer a promising avenue for microbial modulation, providing a variety of antibacterial processes and the ability to alter microbial communities and biofilms (Rani et al., 2021). These natural compounds hold potential as supplementary agents against resistant infections (Rani et al., 2021). Actinomycetes (e.g., *Streptomyces* spp.), fungi (endophytic, filamentous, marine-derived, and mushrooms), and microalgae along with archaea and bacteria are among the major sources of natural antimicrobial compounds (Seal et al., 2018).

The paper highlights several applications of these natural products, including their antimicrobial, antifungal, antibiofilm, and immunomodulatory activities, as well as their utility in agriculture, aquaculture, and the food sector. This paper gathers the current understanding of natural products for microbial modulation, focusing on their antimicrobial activity and broader implications across various sectors, underscoring their potential as a viable solution to antibiotic resistance.

## 2 Sources of natural products and their mechanisms of microbial modulation

### 2.1 Antimicrobial action

The majority of microbial metabolites act at specific target locations and have distinct antibacterial capabilities (Rani et al., 2021).

There is a diverse range of novel antimicrobial compounds such as liamocin oil (from the fungus *Aureobasidium pullulans*) that has antibacterial activity against *Streptococcus* (Price et al., 2017). An antibacterial polypeptide, laparaxin, secreted by *Lactobacillus paracasei* NRRL B-50314 has antibacterial activity against many gram-positive bacteria (Liu et al., 2012).

Food-grade lactic acid bacteria (LAB) are the source of bacteriocins, which are safe and effective against a variety of bacteria along with sporostatic/sporicidal activity against bacterial spores (Egan et al., 2016). Meconium, the earliest stool of a mammalian newborn, is reported to have bacterial species *Enterococci*, *Bifidobacteria*, and *Lactobacilli* and protects mucus of infants from pathogenic species by producing antimicrobial substances (Al Atya et al., 2015).

Microcin, a 21-amino acid polypeptide produced by *Escherichia coli*, has bacteriostatic activity against *Salmonella* Newport ATCC 6962 and members of the Enterobacteriaceae (Gomaa et al., 2017). The bacteria *Lactococcus lactis* and a strain of *Streptococcus uberis* produce a polycyclic antibacterial peptide, which possesses broad-spectrum antibacterial action against a variety of food-spoilage pathogens (Hammami et al., 2015).

Compared to terrestrial bacteria, marine bacteria have numerous unique secondary metabolites as it lives in a more complicated and biologically competitive environment with distinct pressure, temperature, salinity, oxygen, light, and pH conditions (Rani et al., 2021).

A marine bacterium called *Marinomonas mediterranea* was discovered in the Mediterranean Sea near the coast of Murcia and exhibited antagonistic action against *Pseudomonas* sp. *and S. aureus* resistant to ceftazidime and methicillin, respectively (Lucas-Elio et al., 2005). Isatin from the marine bacterium *Pseudoalteromonas rubra* TKJD 22 linked with a marine tunicate, showed antibacterial efficacy against MDR pathogens including MDR *E*. *coli*, *B*. *cereus*, *Micrococcus luteus*, and *B*. *megaterium* (Ayuningrum et al., 2019). A macrolactone named Streptoseomycin from the *Streptomyces seoulensis* A01 demonstrated specific activity against microaerophilic bacteria *Helicobacter pylori* (Zhang et al., 2018). Cyclic peptides such as mathiapeptide A, alkaloids, and sesquiterpenes derivatives named caboxamyxin and mafuraquinocins A and D isolated from bacteria, have antimicrobial properties against clinically resistant bacteria, *Staphylococcus aureus*, methicillin-resistant *Staphylococcus aureus* (MRSA), *Micrococcus luteus*, *Bacillus subtilis*, and *Enterococcus faecalis* (*Ent*. *faecalis*) (Tortorella et al., 2018).

The structure of natural antimicrobial compounds from different microorganisms and their target and mechanism of action are listed in **Table 1**.

**Table 1:** Microbial natural products as antimicrobial agents.

Product	Producer	Active against	Activity	Structure	Reference
Vinaceuline	*Streptomyces* sp. YIM64018	*Penicillium citrinum*, *Gibberella* *zeae*, and *Colletotrichum musae*	Antibacterial activity		Price et al. (2017)
Antimycin A18	Streptomyces		Antimicrobial	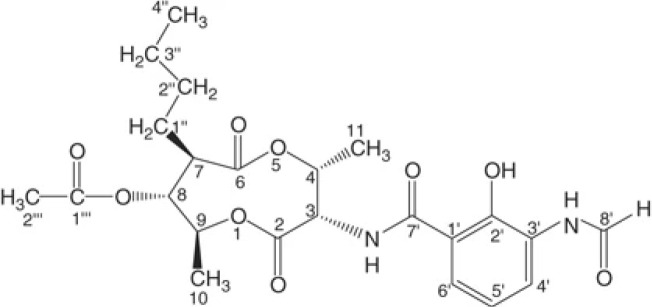	Al Atya et al. (2015)
HalH1	Haloferax mediterranei Xia3	Halobacteriales family members	Change in membrane permeability		Besse et al. (2015)
Lactones	*Phomopsis* sp. YM 311483	*A. niger, Botrytis cinere*, and *Fusarium*	Antimicrobial	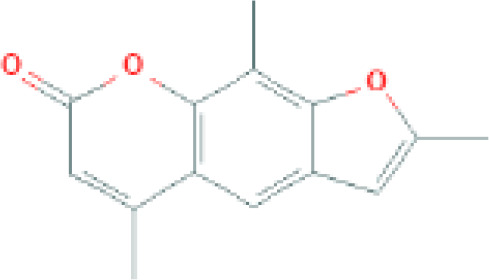	Wu et al. (2008)
Jesterone	*Pestalotiopsis jesteri*	*Pythium ultimum, Phytophthora* *citrophthora, Rhizoctonia solani*, and *Sclerotinia sclerotiorum*	Antimicrobial	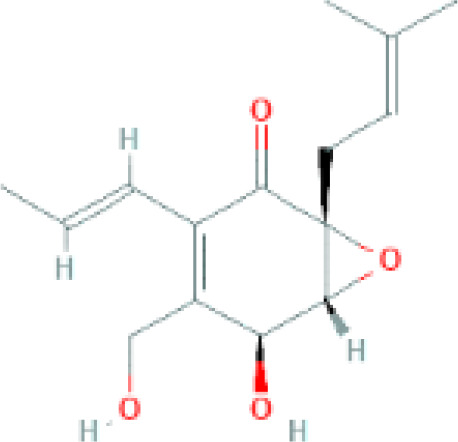	Toghueo et al. (2020)
Penicisteroid A	*Penicillium* *chrysogenum* QEN-24S	*A. niger* and *Alternaria* *brassicae*	Antimicrobial	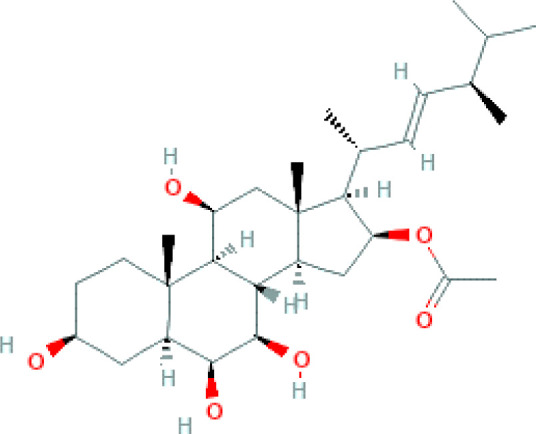	Gao et al. (2010)
Arisugacin K	*P. echinulatum*	*E. coli*	Antimicrobial	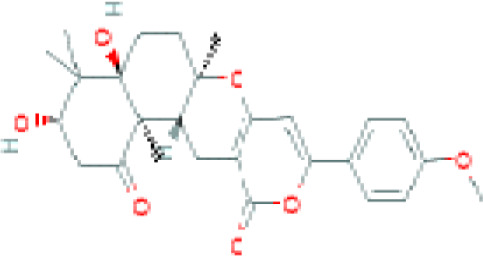	Wang et al. (2021)
Chermesins	*P. chermesinum*	*C. albicans*, *E. coli*	Antimicrobial	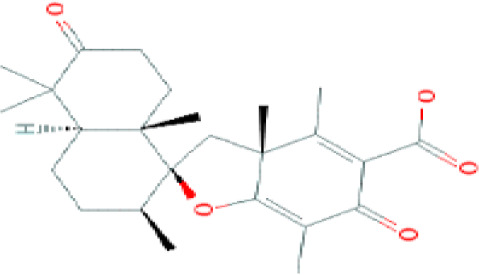	Liu et al. (2016)
Methanolic extract	*Scenedesmus quadricauda*	*S. aureus* and *P. aeruginosa*	Antimicrobial		Arguelles (2018)
Eicosapentaenoic acid	*Phaeodactylum tricornutum*	*Listonella anguillarum*, *Lactococcus* *garvieae*, *Vibrio* spp. and MRSA	Antimicrobial		Debosis et al. (2019)
Hydrophilic extracts	*C. vulgaris*	*Steinernema feltiae*	Antimicrobial		Zielinski et al. (2020)
Unsaturated, saturated long-chain fatty acids	*S. costatum*	*Vibrio* spp., *Pseudomonas* sp., and *Listeria monocytogenes*	Antimicrobial		Alsenani et al. (2020)
Methanolic extracts	*Chlamydomonas reinhardtii*	*A. niger, A. fumigatus, C. albicans*, *S. aureus*, and *E. coli*	Antimicrobial		Ghaidaa et al. (2020)
Marthiapeptide A	*Marinactinospora* *thermotolerans*	*S. aureus, M. luteus, B.* *subtilis, B. thuringiensis*	Antimicrobial	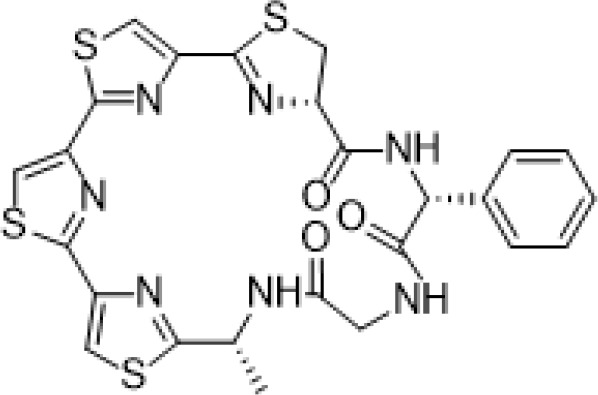	Tortorella et al. (2018)
Desotamide B	*Streptomyces scopuliridis*	*S. aureus, S. aureus*	Antimicrobial	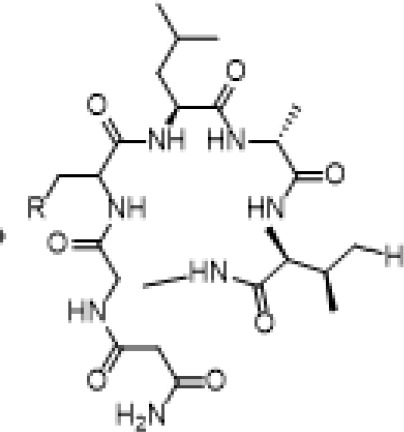	Tortorella et al. (2018)
Marfomycins A, B, E	*Streptomyces* *drozdowiczii*	*M. luteus*	Antimicrobial	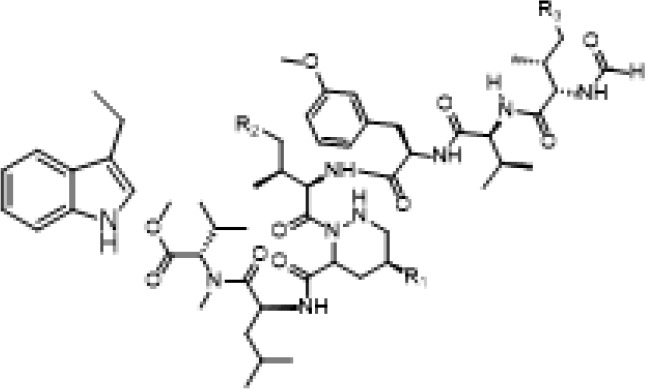	Tortorella et al. (2018)
Caboxamycin	*Streptomyces* sp.	*S. epidermis, S. lentus*, and *B.* *subtilis*	Antimicrobial	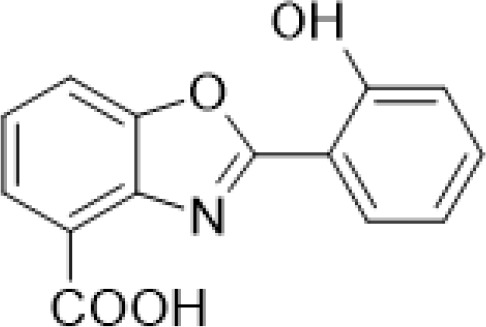	Tortorella et al. (2018)
Marfuraquinocin A, D	*Streptomyces niveus*	Methicillin-resistant *S. aureus*		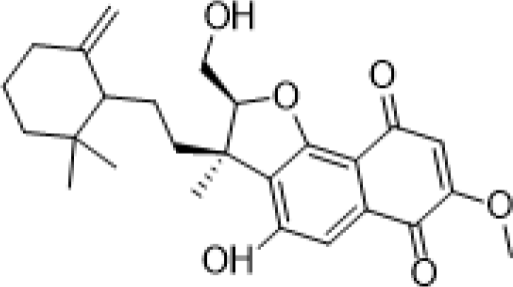	Tortorella et al. (2018)

### 2.2 Antifungal action

Over the past few years, fungal infections have dramatically grown, posing an increasing risk; however, only a few antifungal medications such as polyenes, azoles, and cancidas are available to treat fatal fungal infections. It is necessary to create naturally occurring antifungal medicines with a unique mode of action.

Members of the genus *Bacillus* produce a vast array of biologically active molecules. Some potential antifungal compounds isolated from these bacteria are mycobacillins, iturins, plistatins, bacillomycins, surfactins, mycosubtilins, fungistatins, zwittermicin, and macrolactins (Sansinenea, 2020).

The secondary metabolites of *Bacillus* sp. producing antimicrobial lipopeptides and other compounds function as antifungals against many phytopathogens infecting agricultural crops (Salazar et al., 2020).

Ballad Plus and Sonata, two commercial products from Bayer CropScience based on *B*. *pumilus* (strain QST 2808), generate an antifungal amino sugar molecule that interferes with cell metabolism and breaks down cell walls, killing plant infections (Serrano et al., 2013).

Ieodoglucomide and ieodoglycolipid separated from the ethyl acetate extract of a marine-derived *Bacillus licheniformis* have an antifungal activity against the human pathogen *Candida albicans* as well as the plant pathogens *Colletotrichum acutatum* and *Botrytis cinerea* (Rani et al., 2021). Similarly, janthinopolyenemycin A and B polyketides isolated from the proteobacterium *Janthinobacterium* sp. prevented the growth of *C*. *albicans* (Anjum et al., 2018).

The peptide nucleosides isolated from *Streptomyces cacaoi* inhibited the enzyme chitin synthase leading to the prevention of biosynthesis of chitin in insects (Arakawa, 2003).

The peptide HP (2–20), derived from the N-terminal sequence of *Helicobacter pylori* ribosomal protein L1 (RPL1), has an nematicidal activity against the eggs and worms of *Caenorhabditis elegans*—disrupting the egg shell and the cell membrane structurally (Jang et al., 2004). *Streptomyces* species, the aerobic gram-positive branching bacilli yields some antifungal compounds including nystatin, phthoxazolin A, faeriefungin, butyrolactols A and B, sultriecin, polyoxin, and dunaimycins (Amaning Danquah et al., 2022).

Antifungal agents are also classified by their mode of action. The fungal cell wall is composed of glucan, chitin, and mannoproteins along with sphingolipids, in relatively small proportions. Antifungal agents acting on these major targets for the development of novel antifungals are inherently selective.

The inhibition of sphingolipid synthesis also results in the inhibition of growth and cell death.

Three key enzymes serine palmitoyltransferase, ceramide synthase, and inositol phosphoceramide (IPC) synthase are involved in the sphingolipid synthesis pathway and have been targeted for the development of novel antifungals. Sphingofungins, lipoxamycin, and viridiofungins inhibit serine palmitoyltransferase. Fumonisin B1 and australifungin inhibit ceramide synthase, and aureobasidins khafrefungin and rustmicin inhibit IPC synthase. A novel compound, minimoidin indirectly inhibits sphingolipid synthesis by blocking the fatty acid elongation pathway.

Fungal soluble factors EF3, required only by fungal ribosomes, and EF2, essential for protein synthesis, are also used as targets for antifungal drug discovery (Mandala et al., 2000; Vicente et al., 2003).

The structure of antifungal compounds and their source, target organism, and mechanism of action are listed in **Table 2**.

**Table 2:** Microbial natural products as antifungal agents.

Compound		Producing species	Mechanism of action	Structure	Reference
Lipopeptides	Echinocandin B Pneumocandins	*Aspergillus nidulans* *Glarea lozoyensis*	Inhibitors of glucan synthesis		Nyfeler and Keller-Schierlein (1974)
Acidic terpenoids	Efumafungin Ascoteroside	*Hormonema* sp. *Ascotricha amphitricha*	Inhibitors of glucan synthesis		Peláez et al. (2000)
Sphingofungins		*Aspergillus fumigatus*	Serine palmitoyltransferase inhibitors	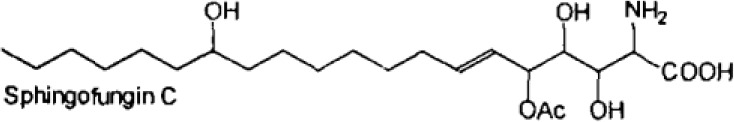	Peláez et al. (2000)
Lipoxamycin		*Streptomyces* sp.	Serine palmitoyltransferase inhibitors	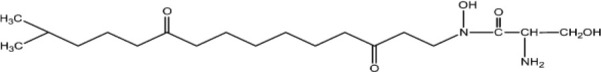	Vicente et al. (2003)
Viridiofungins		*Trichoderma viride*	Serine palmitoyltransferase inhibitors	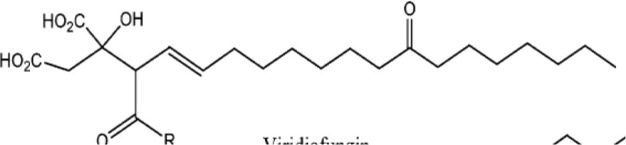	Vicente et al. (2003)
fumonisins		*Fusarioum moniliforme*	Ceramide synthase inhibitors	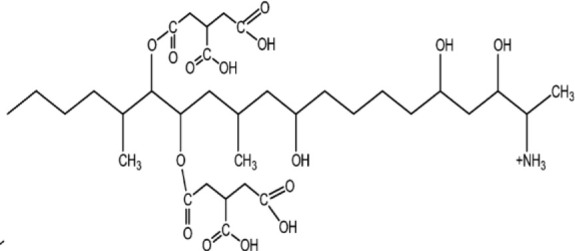	Wang et al. (1991)
Australifungin		*Sporormiella australis*	Ceramide synthase inhibitors	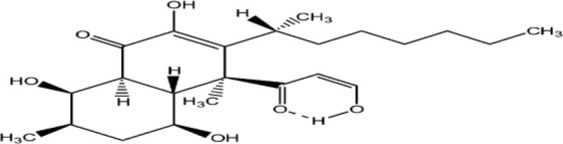	Mandala and Harris (2000)
Aureobasidins		*Aureobasidium pullulans*	IPC synthase inhibitors	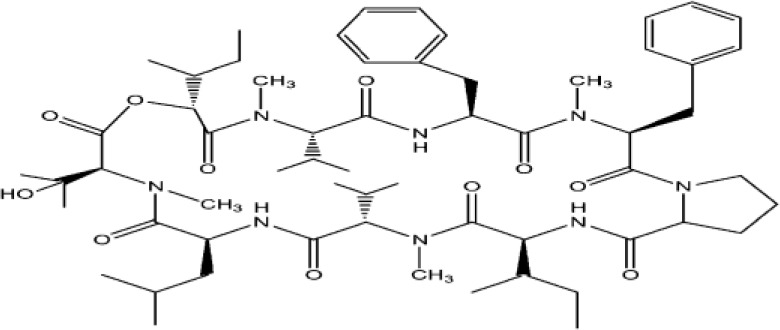	Mandala and Harris (2000)
Minimoidin		*Sporomiella minimoides*	Fatty acid elongation inhibitors	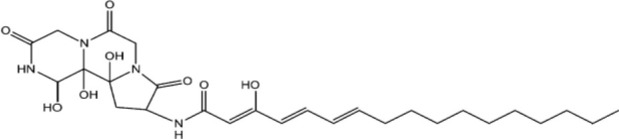	Vicente et al. (2003)
Sordarin		*Sordaria araneosa*	Protein synthesis inhibitors	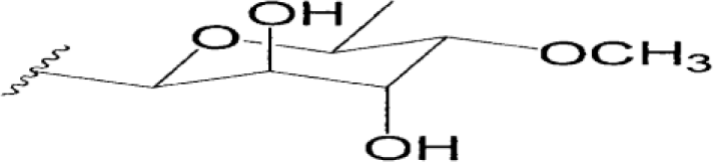	Peláez et al. (2000)

### 2.3 Antiviral activity

Virus infections, being a major reason for morbidity and mortality, represent significant threats to health care at the global level (Araujo et al., 2022).

Secondary metabolites from microorganisms are considered promising substances for the development of antiviral compounds. Some species of *Pseudomonas* and *Burkhoderia* produce anionic biosurfactants Rhamnolipid, which have shown activities against microorganisms, biofilms, tumors, and oxidation reactions (Herzog et al., 2020; Thakur et al., 2021). It also interacts with viral lipid membranes and alters viral membrane glycoproteins in Herpes simplex virus 1 and 2 (HSV-1 and HSV-2) and bovine coronaviruses (Jin et al., 2021). Surfactin from *B. subtilis* inhibits membrane fusion in enveloped viruses like HSV-1 and HSV-2 (Yuan et al., 2018). Rhamnolipids (M15RL) produced by the Antarctic bacterium, *Pseudomanas gessardii* M15 exhibited a high antiviral activity against severe acute respiratory syndrome coronavirus 2 (SARS-CoV-2) along with other members of the Coronaviridae and Herpesviridae families (Giugliano et al., 2021). Sophorolipids from *Candida bombicola* have virucidal properties against human immunodeficiency virus (HIV) (Borsanyiova et al., 2016).

### 2.4 Antibiofilm activity and quorum sensing inhibition

Microbial products either work alone or synergistically to prevent the production of biofilms by creating less selective pressure and by developing resistance (Raj and Thomas, 2021; Panigrahy and Kumar, 2019). Microbial anti-biofilms can resist harsh environmental conditions and maintain their efficacy and activity without being toxic to the host (Asadi et al., 2019). Antibiofilm-producing microbes suppress cell attachment by interfering with the forces (electrostatic attraction, sedimentation, Brownian movements, and Van der Waals force of attraction) and adhesion by preventing the production of alginate and exopolysaccharide (Lee et al., 2013). Additionally, they hinder the formation of extracellular matrix, inhibit cell survival and division, stop biofilm development, deprive substrates, and disrupt the quorum-sensing mechanism by downregulating molecules including autoinducer type 2 and acyl homoserine lactone (Silva et al., 2020). The antibiofilm compounds may include bioactive compounds, biosurfactants, antimicrobial peptides, or enzymes (Rani et al., 2021; Hussaini et al., 2024).

Carolacton, produced by the myxobacterium *Sorangium cellulosum*, exhibited an anti-biofilm activity against *Streptomyces mutans* by affecting numerous regulatory systems of the organism (Kunze et al., 2010). Macrotetrolides (monactin, dynactin, and trinactin), isolated from *Saccharomyces cerevisiae*, and polymers produced by *Trichosporon montevideense* showed anti-biofilm activities against *C*. *albicans* (Tebbets et al., 2013; Ceresa et al., 2016).

Bacterial biofilms formed by isolates *S*. *aureus*, *B. subtilis*, and *Ent*. *faecalis* were inhibited by compounds (Diterpene and indole alkaloids) produced by *Neosatorya fischeri* KUTC 6344 (Eamvijarn et al., 2013). The biofilm produced by methicillin-resistant *S*. *aureus* (MRSA) was inhibited by cytosporone E isolated from *Leucostoma persoonii* (Beau et al., 2012).

By interfering with the RhlR and LasR proteins that control the expression of virulence genes in *Pseudomonas aeruginosa*, patulin and penicillanic acid isolated from *Penicillium coprobium* and *P*. *radicicola*, respectively, exhibited quorum-sensing inhibitory activities (Rasmussen et al., 2005). A lantibiotic, gallidermin produced by *Staphylococcus gallinarum* inhibited biofilm formation by *S*. *aureus* and *S*. *epidermidis* (Saising et al., 2012).

Amino acids and their derivatives derived from microorganisms have also been reported to possess anti-biofilm activities. Biofilms produced by *S*. *epidermidis* and *S*. *aureus* were inhibited by the dipeptide isolated from a sponge associated with *Penicillium* sp. and lovastatin from *Penicillium commune*, respectively (Scopel et al., 2013; Diblasi et al., 2015). Amino acid antibiotic isolated from *Paenibacillus* sp*ecies* 139SI exhibited an anti-biofilm activity against both gram-positive and gram-negative bacterial isolates by inhibiting cell – cell interaction and cell–surface attachment (Alasil et al., 2014).

EPS-273, an extracellular polysaccharide obtained from the marine bacterium *Pseudomonas stutzeri* 273, prevents biofilm formation in *P*. *aeruginosa* by decreasing the production of pyocyanin (a virulence factor) (Wu et al., 2016). Another two polysaccharides produced by *P. aeruginosa* PAO1, namely Pel and Psl, reduced biofilm formed by *S. epidermidis* (Pihl et al., 2010). The sulfated polysaccharides produced by *Chlamydomonas reinhardtii* disrupted biofilm formed by *Salmonella enterica* and *Vibrio harveyi* by degrading the eDNA component of the EPS matrix (Vishwakarma and VL, 2020).

Biosurfactants, a heterogeneous group of surface-active amphiphilic compounds produced by diverse groups of microorganisms, possesses antifungal, antibacterial, and anti-biofilm properties (Paraszkiewicz et al., 2021).

Biosurfactants alter the ability of cells to adhere to surfaces by decreasing the hydrophobicity of the cell surface, rupturing the membrane, blocking the electron transport chain, and reducing the energy requirements of the cell (Satputea et al., 2016). They also prevent pathogenic organisms from forming biofilms and hence, serve as a good coating material for medical implants such as bone implants and urinal catheters without using synthetic drugs (Raj and Thomas, 2021). Biosurfactants from *Lactobacillus gasseri* inhibited biofilm formation in some strains of methicillin-resistant *S. aureus* (MRSA) (Giordani et al., 2019).

Lipopeptide biosurfactants produced by *Acinetobacter junii* B6 disrupted biofilm production by *Proteus mirabilis*, *S*. *aureus*, and *P*. *aeruginosa* (Ohadi et al., 2020). They also regulate quorum sensing and the motility of bacteria (Sharma et al., 2021). The ability of biosurfactants produced by *Cobetia* sp. blocked quorum sensing by interfering with the lipophilic signals involved in intercellular communication, ultimately leading to the repression of genes involved in biofilm formation (Ibacache-Quiroga et al., 2013).

The antibiofilm property of lipopeptide biosurfactant produced by *B. tequilensis* strain SDS21 eradicated >99% of the biofilms formed by *E*. *coli*, *P*. *aeruginosa*, *S*. *aureus*, *S*. *epidermidis*, *Salmonella typhi*, and *Salmonella typhimurium* on different types of surfaces (Singh and Sharma, 2020). *S. aureus* biofilms were inhibited by mannosyl erythritol lipids isolated from *Pseudozyma aphidis* DSM through the disruption of bacterial adhesion to surfaces.

Pontifactin, a biosurfactant produced by a marine bacterium *Pontibacter korlensis*, exhibited an anti-biofilm activity by increasing or altering the permeability of bacterial membranes against isolates of *S. aureus*, *Salmonella typhi*, *Vibrio cholerae*, and *B. subtilis* (Balan et al., 2016).

Antimicrobial peptides (AMP) from microorganisms prevent the formation of biofilm or eradicate mature ones by electrostatic interaction with membrane phospholipids. AMPs are mostly cationic amphiphilic compounds but anionic and neutral peptides also exist (Mishra et al., 2020).

An AMP isolated from *Bacillus* species P34 eradicated biofilms formed by *S. aureus* and *Ent. faecalis* (Costa et al., 2018). A post-translational modified peptide, microcin-B17 from *E. coli* exhibited an anti-biofilm activity by inhibiting the division and survival of bacterial cells (Asma et al., 2022). Certain AMPs also inhibit biofilm formation by penetrating deep into the biofilm and interfering with the integrity of lipopolysaccharides of the bacterial cell leading to the disruption and killing of the bacteria (Roy et al., 2018).

Actinobacteria, the most dominant phylum in the bacterial domain, are a prolific source of numerous bioactive compounds used in the pharmaceutical, agricultural, biotechnology, and food industries. Not only the genus Streptomyces but also non-Streptomyces or rare Actinobacteria show anti-biofilm activities against a wide range of bacteria (Azman et al., 2019).

### 2.5 Immunomodulatory interaction

Bacterial natural compounds exhibit anti-inflammatory, drug-like activity by modulating cytokines. Because of faster growth compared with other microorganisms, bacteria are recommended as sources of anti-inflammatory inhibitors for large-scale production (Jenab et al., 2020).

Antimicrobial peptides have shown immunostimulatory functions either by membrane-active or non-membrane active mechanisms (Naiel et al., 2023). In membrane-active mechanisms, pores are induced on the entire cell surface membrane following their electrostatic reaction, consequently performing the discharge of cellular constituents and sudden cell death (Sengupta et al., 2008). In non-membrane-active mechanisms, the steps required for protein/DNA/enzyme activity or cell division are inhibited. During immunostimulatory action, cytokine production is triggered, leading to the improvement of cell mediated and humoral immunity.

Surfactin, a bacterial cyclic lipopeptide produced by *B. subtilis*. prevents the formation of inflammatory agents like IL-1β and iNOS, along with a decrease in TNF-α and nitric oxide levels. It also has anti-inflammatory properties by reducing the activation of nuclear factor-κB (NF-κB) involved in cell-signaling pathways (Zhang et al., 2015).

Other cyclic lipopeptides like fengycin, and iturin lipopeptides produced by *B. subtilis* have shown anti-inflammatory properties, along with interactions with biofilms, anti-fungal, anti-tumor, anti-virus, and anti-platelet activities (Zhao et al., 2017). The interleukin-4 (IL-4) and interleukin-5 (IL-5) levels decreased to normal after administration of branched gluco galactan, 2-1-Kefiran produced by lactic acid bacteria in BALB/c mice stimulated with ovalbumen (Kwon et al., 2008). 2-2-Exopolysaccharide (EPS) from the probiotic spore-forming bacterium *B. subtilis* inhibited T-cell activation and controlled T-cell-mediated immune responses in various inflammatory diseases (Hsieh and Allen, 2020).

The secretion of pro-inflammatory cytokines (TNF-α, IL-1β, IL-8, IL-2, and IFNγ) and anti-inflammatory cytokines (IL-4 and IL-10) by human peripheral blood mononuclear cells (PBMC) was increased by secondary metabolites isolated from *Bacillus* sp. from Neogene permafrost (Todorov, 2009). More detailed descriptions of various immunomodulatory compounds are listed in **Table 3**.

**Table 3:** List of immunomodulatory agents from microbes.

Bacterial bio-active compound	Source	Activity	Reference
Biosurfactant	*Bacillus licheniformis*	IL10, TGF TNF-α and IL1β	Soria-Mercado et al. (2012)
Bacteriocin	*Lactobacillus rhamnosus*	CRP, IL 6	Rund et al. (2000)
Surfactin	*B. subtilis*	TNF-α and nitric oxide	Marinelli et al. (2015)
Bacteriocin (nisin)	LAB bacteria	Downregulation of lung Th2 response by increasing IFN-γ and reducing IL-4 and IL-13	Lewies et al. (2019)
Arachidonic acid	*Psychroflexus tarquis* *Psychroflexus pacifica*	inhibition of NO and TNF-α	Kook et al. (2019)
Valeric acid (pentanoic acid) Caproic acid (hexanoic acid)	Megasphaera massiliensis MRX0029 Ruminnococcaceae CPB0	Repression of IFN-γ, IL-10, IL-1β, and TNF-α	Mikelsaar et al. (2016)
Kefiran (branched glucogalactan)	Lactic acid bacteria	IL-4 and IL-5 levels reduced to normal levels	Jenab et al. (2020)
Dopamine (from dietary substrate)	Enterococcus faecium	Modulation of the immune system	Jenab et al. (2020)
*Butanol extract*	*Bifidobacterium adolescentis*	Boosting of TNF-α and NO	Yu et al. (2018)
Camporidine A	Strain *Streptomyces* sp. STA1	Nitric oxide production suppressed	Mace et al. (2019)

## 3 Application of microbial products

### 3.1 Applications in agriculture

To meet the demands of the growing population, crop yields must rise in parallel. Although chemical fertilizers can accomplish the goal, excessive and ongoing use leads to health issues, pest resistance, and ecological harm (Youssef and Eissa, 2014). Chemical fertilizers can be replaced by biofertilizers by increasing the supply or availability of primary nutrients to the host plant. Natural processes such as atmospheric nitrogen fixation, phosphorus solubilization, and plant growth stimulation through the synthesis of growth-promoting substances helps biofertilizers add nutrients (Malusà et al., 2016).

Plant growth-promoting rhizobacteria (PGPR) are a group of bacteria that colonize the roots of plants and enhance growth by producing plant hormones or secondary metabolites (Keswani et al., 2019). There are many PGPR such as *Rhizobia*, *Mycorrhizae*, *Azospirillum*, *Bacillus*, *Pseudomonas*, *Trichoderma*, and *Streptomyces* species that control diseases, induce systemic resistance, or change physicochemical interactions with plants (Backer et al., 2018). *Bacillus* can act directly either by obtaining nutrient supply such as nitrogen, phosphorus, potassium and minerals or by modulating plant hormone levels. It secrets antagonistic substances to inhibit or induce resistance to plant pathogens indirectly (Sansinenea, 2019).

The soil quality, soil health, growth yield, and quality of crops improved by beneficial microorganisms through the production of bioactive substances such as hormones and enzymes. These microorganisms promote plant growth by controlling soil diseases and accelerating decomposition of lignin materials in the soil (Keswani et al., 2020).

The entomopathogenic bacterium *B. thuringiensis* and *Bacillus* spp. such as *B. amyloliquefaciens*, *B. licheniformis*, *B. pumilus*, and *B. subtilis* have been widely used as a natural biopesticide (Sansinenea, 2012). Several bacteria and fungi present ubiquitously in different soils assist plant growth by mobilizing insoluble forms of potassium (Ortiz and Sansinenea, 2021).

Recently, formulations of biological control organisms have been used commercially to control diseases in agricultural and horticultural crops. The application of chemical fungicides to control post-harvest diseases is restricted due to safety concerns and development of pathogen resistance (Janisiewicz and Korsten, 2002). The spores of the naturally occurring soil bacteria *B. velezensis* or *B. atrophaeus* have been commercialized as biofertilizers under the name RhizoVital by AbiTEP GmbH (Chowdhury et al., 2013).

The application of modified antimicrobial peptide (AMP) showed strong resistance to late blight and pink rot phytopathogens, in addition to the bacterial pathogen *Erwinia carotovora* in potato (Osusky et al., 2004). A commercially formulated product, Avogreen from *B. subtilis* B246 is used as a biocontrol agent against anthracnose caused by the fungus *Colletotrichum gloeosporioides* (Demoz and Korsten, 2006).

The AMPs against bacterial and fungal plant pathogens were assessed to screen transgenic crops (Keymanesh et al., 2009).

The expression of mammalian AMP cecropin P1 in transgenic tobacco resulted in increased resistance to phytopathogenic bacteria *Pseudomonas syringae*, *Pse. marginata*, and *Erwinia carotovora* (Zakharchenko et al., 2005).

The significantly enhanced resistance in transgenic tobacco against the fungal pathogen, *Colletotrichum destructivum*, and the bacterial pathogen *Pseudomonas syringae*, was achieved by the expression of AMP MSI-99 via the chloroplast genome (DeGray et al., 2001).

The harmful fungal pathogen *V*. *dahliae* occurs in potatoes inhibited strongly by Alfalfa antifungal peptide (alfAFP) isolated from the seeds of *Medicago sativa* (Gao et al., 2000).

Tachyplesin, an AMP isolated from the hemocytes of the *Tachypleus tridentatus*, has been evaluated as a potential candidate for the inhibition of Sclerotinia disease in sunflower (Lu, 2003). The use of attacin E, an AMP that originated from *Hyalophora cecropia*, resulted in significant resistance to the bacteria *Erwinia amylovora*, which causes fire blight disease.

### 3.2 Application in aquaculture

AMPs are significant substances that have been shown to have antibacterial, antifungal, antiviral, and antiparasitic properties against a variety of fish-associated pathogens such as viruses, bacteria, fungi, and parasites (Brogden, 2005).

The mortality rate induced by the *Vibrio harveyi* infection was reduced with AMPs by promoting growth, serum antioxidant status, and innate immunity (Gyan et al., 2020). Another liver-expressed AMP-2 (LEAP-2) isolated from *Trachinotus ovatus* strongly suppressed *Streptococcus agalactiae* and *Edwardseilla tarda*, boosting the immunity (Lei et al., 2020).

### 3.3 Application in the food industry

Numerous substances are produced by bacteria, including fermentation by-products such as organic acids, hydrogen peroxide, and diacetyl, as well as bacteriocins and other antagonistic substances that can inhibit the growth of pathogenic microorganisms responsible for spoilage and disease in the food industry (Tiwari et al., 2009).

Bacteriocins, a proteinaceous antibacterial compound that inhibits undesirable bacterial growth in foods, help maintain the freshness and quality of food products over a longer period. They are cationic peptides that display hydrophobic or amphiphilic properties, and in most cases, the target of their activity is the bacterial membrane. A large number of bacteriocins have been isolated and characterized from lactic acid, and they act as an extra barrier that can keep food safe even when storage or transport conditions are not optimal (Parada Fabián et al., 2025).

The FDA has approved the use of nisin, a naturally occurring AMP with a narrow range of activity, as a food preservative. It is produced during fermentation by specific strains of the lactic acid bacterium *Lactococcus lactis* and quite effective against a variety of gram-positive bacteria (Hancock and Lehrer, 1998). In addition to preventing microbial growth in beef, sausages, liquid whole eggs, ground beef, and poultry, it is also utilized in the cheese industry to restrict the growth of *Clostridium* spp. It also suppresses the subsequent growth of *Listeria monocytogenes* in ready-to-eat (RTE) meat products (Tiwari et al., 2009). Moreover, it effectively controls *Alicyclobacillus acidoterrestris* in fruit juices (Komitopoulou et al., 1999). Nisin also reduces the growth of *S. aureus*, *L. monocytogenes*, and the spores of *C.* sp*orogenes* in cold-packed cheese spreads. It also inhibits *Clostridium*-related butyric acid fermentation by inhibiting the growth spores of clostridia like *Clostridium ttyrobutyricum* (Lahiri et al., 2022).

Reuterin (β-hydroxypropionaldehyde) is a water-soluble nonproteinaceous broad-spectrum antimicrobial compound produced by some strains of *L. reuteri* effective against gram-negative and gram-positive bacteria, yeasts, and filamentous fungi. It is active over a broad range of pH and is resistant to proteolytic and lipolytic enzymes (El-Ziney et al., 1999).

Pediocins are thermostable, amphipathic proteins with a loop formation formed by three β-sheets. It is effective against both spoilage and pathogenic organisms, including *L. monocytogenes*, *Ent. faecalis*, and *S*. *aureus* over a wide range of pH values. Pediocin is produced by strains of *Pediococcus acidilactici* and *P*. *pentosaceus* used in fermented sausage production (Anastasiadou et al., 2008). It is also used as a preservative in vegetable and meat products with high activity against *Listeria* species (Parada Fabián et al., 2025).

## 4 Challenges and future prospectus

Natural products from microbes replace traditional antibiotics and become effective molecules against infectious diseases. However, some research questions such as the efficacy and applicability of these products under field trials limit their use. A more in-depth study on the effects of these extracts/compounds is needed to investigate their mechanism of action. The antifungal, antiviral, and antiparasitic effects should also be validated with *in vivo* and *in vitro* trials to understand their precise mode of action. Product formulation, extrinsic storage parameters, and intrinsic product parameters also require further study.

Since the use of antibiotics in food is inhibited, the bacteriocin used as a food preservative should be declared as generally recognized as safe (GRAS). The manufacturing process, quantification, and standardization assays, with toxicological data and the fate of the molecule after consumption should be documented prior to approval (Lahiri et al., 2022). The chemical composition of bacteriocins should be identified by using standard biochemistry and molecular techniques (Parada Fabián et al., 2025). Regulatory approval in various regions is crucial for the commercialization of bacteriocins in the global market, ensuring safe, natural, and sustainable solutions.

Multiple advanced approaches such as culture strategies, genomics mining, and artificial intelligence (AI), along with genome editing, ribosome engineering, precursor engineering, mutagenesis, and overexpression of structural genes, can produce natural products and pharmaceuticals in microbial systems efficiently by overcoming these hurdles (Yang et al., 2025; Pham et al., 2019). Engineering strategies and recombinant DNA technologies can activate silent and cryptic biosynthetic gene clusters (BGCs), leading to increased production of microbial natural products and recombinant proteins (Madden et al., 2025). Artificial intelligence (AI) can scan biological sequences to identify potential AMPs along with prediction of their activity and toxicity (Szymczak et al., 2025). By harnessing the power of these engineered technologies, we can design novel, effective, and safe compounds that open new frontiers in the fight against AMR.

## 5 Conclusion

Natural products from microbes offer a variety of antibacterial mechanisms, can alter microbial communities and biofilms, and have potential as supplementary agents against resistant infections. To achieve their clinical promise, however, significant translational work—standardized formulations, toxicity testing, and rigorous clinical trials—is needed. Natural antimicrobials may have a significant impact on the treatment of infectious diseases in the future by fusing traditional knowledge with various engineered technologies.
